# Personality and Attitudes Confronting Death Awareness During the COVID-19 Outbreak in Italy and Spain

**DOI:** 10.3389/fpsyt.2021.627018

**Published:** 2021-02-04

**Authors:** Mike Murphy, Carmen Moret-Tatay

**Affiliations:** ^1^School of Applied Psychology, University College Cork, Cork, Ireland; ^2^Faculty of Psychology, Universidad Católica de Valencia San Vicente Mártir, Valencia, Spain; ^3^Il Dipartimento di Neuroscienze Salute Mentale e Organi di Senso, Sapienza Università di Roma, Rome, Italy

**Keywords:** COVID-19, death awareness, cluster analysis, COVID-19 outbreak, pandemic, personality, fear, death

## Abstract

Italy and Spain are two representative examples on strict lockdown last March 2020, also suffering a high rate of mortality in Europe. The aim of this study is to examine their attitudes confronting death awareness during the Covid-19 outbreak. Moreover, Personality was also considered. Different sociodemographic, *in situ* questions related to attitudes and the brief Big Five of Personality were employed in a cross-sectional design. The main results suggested that Personality traits were stable across countries. A relationship was found between Fear to contagious diseases and Neuroticism and other attitudes during the Covid-19 outbreak, and two different clusters were identified with regards to attitudes, however these did not differ on Personality. Finally, a Cluster group, Neuroticism, Age and Sense of belonging to the Country did predict Fear to contagious diseases. Of note, no differences were found across countries during grief.

## Introduction

Humans are an adaptable species. However, natural disasters such as the current Covid-19 outbreak might cause people to see their normalcy completely altered by not being able to socialize as we are used to, as well as, losing contact with our relatives or, even in extremes cases, losing someone they love (1, 2). In this way, it seems important to remark that people have been exposed to death at close quarters during the Covid-19 outbreak, whether it be from a family member, a neighbor, an acquaintance, including children who may not understand what happens to those people or health workers suffering from post-traumatic stress disorders (3). Given that different or common country measures were applied worldwide, studies across different nations allow, not only the understanding of our own nation externally, but also other internal processes, where, to some extent, the country in which they are born (4) and therefore the human expression of pain is influenced by the product of culture, customs or symbols among others (5, 6).

Resolving grief often involves cultivating bonds of emotion and meaning with death (7). Cross-cultural studies of grief can be applied to many levels of human life: from a biological level (8) to a linguistic ones (9), into of a vast range of possibilities that involve the meanings and the use of words that West countries use as grief and mourning. However, the current situation might be surely accompanied by other variables, most of them attitudes and fears into a scenario full of uncertainties. First, the fear on an economic crisis of greater impact than the one occurred in 2008 (10). Other variables such as social media misinformation, political polarization, country measures, and social inequities or vulnerabilities, among others, are of interest to shed light on both internal and external anxiety states (11–13). Not surprisingly, the pandemic has raised many issues of debate that involves a large body of disciplines in our society.

As expected, recent studies have also report that this situation will have an important psychological impact on people (14, 15). However, little is known in relation to the attitudes developed in those who has directly suffered the loss of a loved one and close to them, during the pandemic onset. According to the literature (16), circumstances surrounding death in the era of Covid-19 outbreak incorporate multiple and indirect traumatic characteristics, such as multiple deaths in families, social restrictions that prohibit visiting relatives in hospitals or intensive care units causing feelings of guilt, feelings of isolation and precarious socio-economic and living conditions as a result of the pandemic which will in turn be linked to mourning complications.

How personality affects health behaviors is well-recognized in the literature through a large body of research on the Big Five model (17–19). Moreover, a mediational study was carried out on Personality and fear of death among young adults, being neuroticism positively correlated to death anxiety (20). However, authors did not report if participants were if they had been closed to someone's death because of Covid-19 virus. In this way, the aim of this study is to focus in this profile, in order to examine their attitudes confronting death awareness during the Covid-19 outbreak. Moreover, to examine different profiles according to country of origin and residence, attitudes confronting mourning processes, and Personality. Focusing on mortality rates in Europe, two representative examples, who have also suffered strict lockdown last March 2020, are Italy and Spain (21, 22). For this reason, these populations were selected. We hypothesis that Personality is stable across countries in the mourning process. For previous results, we expect that Neuroticism predict fear to contagious diseases in participants who have experienced a loss. Lastly, we hypothesized that different profiles can be identified regarding attitudes during grief, and these are independent to country of origin and residence.

## Methods

### Participants

Inclusion criteria are described as follows: (i) the participants had to be over 18 years old, (ii) to have experienced the loss of someone's close because of Covid-19 virus, (iii) be a resident of one of the three selected countries, and in addition to showing their consent according to the Declaration of Helsinki. If this consent was not accepted, the process did not continue. Participation was completely voluntary and could be withdrawn at any time. In addition, there was also the option of not answering some demographic questions if they did not wish to do so. The information provided by the participants was completely anonymous, where neither the name nor the IP address is recorded so that it cannot be traced in any case.

### Procedure

Data collection was collected online through Qualtrics software, which was distributed by different communication applications. This study was approved by the ethics committee of the UCC School of Applied Psychology Ethics Committee on April 6, 2020. The study was carried out in April 2020, were the Italian measures were well-established, and the Spanish one almost started after middle Mach. During April and May 2020, when data was recruited, citizens should only leave their homes for necessities.

### Materials

Sociodemographic data, as well as list of questions developed for the Covid19 outbreak situation, and specific questioners were employed for the current research, which were listed as follows:

*In situ* questions related to Covid19 outbreak, developed for the propose of the current study, and answered in a 5 likert point scale:

- I fear people with contagious diseases
- I think my sense of belonging to Italy/Spain has increased since the outbreak began
- I am concerned about the economic impact that this pandemic may have in my country
- I consider that I have correctly informed myself about Covid-19
- Self-quarantine is necessary for the good of others
- Self-quarantine is necessary for my own good.

The Big 5 personality inventory was used to assess personality (BFI-10) developed for several countries, such as the Italian and the Spanish one (23). It consists of 10 reagents with a Likert type scale of five values ranging from 5 = complete agreement, 4 = agreement, 3 = neither agreement nor disagreement, 2 = disagree, 1 = strongly disagree, this version is an abbreviated version of the Inventory of the Five Major personality factors (24). The value of Cronbach's Alpha average in the literature was 0.75.

### Design and Analysis

This is a cross-cultural study conducted on an incidental sample. Cases with more than 10% missing were not considered. We imputed missing values through the SPSS method for multiple imputations to produce a new data set without missing data. First, a descriptive approach was carried out. Normality and homogeneity analyses of data were developed, prior to the analyses. Secondly, a relational analysis was carried out, a cluster analysis and a linear regression was performed, in order to make predictions about the variables of interest.

## Results

As can be seen in **Table 4**, both Sense of Belonging and Neuroticism were significant unique predictors of Fear of contagious diseases. According to the specific inclusion criteria, “to have experienced the loss of someone's close because of Covid-19 virus” a total of 75 Spanish resident respondents over 371 (20.2%) were selected in the current study, while a total 42 over 324 (13%) for the Italian sample. However, to better address any bias related to culture in the sample, 5 participants were excluded from the previous number because their nationality was different from the country of residence. The final sample was composed by 72 Spanish respondents living in Spain, and 40 Italian respondents, living in Italy.

For the Spanish sample, 26.4% were men and 73.6% women, while the Italian 30% men and 70% women. With regards to Education the Spanish sample referred a 23.6 of some Primary and complementary School, 41.7 for Secondary School and 34.7% for Higher or further Education, while the Italian Sample was divided into a 17.5% for Secondary School an 82.5% for Higher or further Education. In terms of occupation, the Spanish sample reported the following percentages: (i) 26.7% were self-employed or an employee in an essential service, (ii) 25.3% were working from home, (iii) 8 % remained employed but could not carry out their work, (iv) 4 % were retired, (v) 2.7 % were homemaker, (vi) 26.7 % were full-time student, (vii) 2.7% were unemployed (beginning before the Covid-19 outbreak), (viii) 4 % were unemployed (beginning during the Covid-19 outbreak). The Italian sample was also described as follows: (i) 16.7% were self-employed or an employee in an essential service, ii) 16.7% were working from home, (iii) 11.9 % remained employed but could not carry out their work, (iv) 2.4 % were homemaker, (v) 45.2 % were full-time student, (vi) 4.8 % were unemployed (beginning before the Covid-19 outbreak), (vii) 2.4% were unemployed (beginning during the Covid-19 outbreak). Other descriptive analyses were carried out in the variable of interest and depicted in Table 1 across countries. Moreover, a student's *t*-test was carried out for independent groups. Conscientiousness showed higher scores for Spain than Italy reaching the statistical level: (*p* < 0.001; Cohen's d′ = 0.69).

**Table 1:** Descriptive analysis across the Spanish and the Italian samples.

	**Group**	**Mean**	**SD**	***p***
Age	Italy	29.88	10.70	0.045
	Spain	34.97	14.21	
People I live with	Italy	3.24	3.97	>0.05
	Spain	2.64	2.58	
Fear contagious diseases	Italy	5.38	2.76	>0.05
	Spain	5.43	2.84	
Sense of belonging	Italy	4.93	2.96	>0.05
	Spain	4.43	2.80	
Economic impact	Italy	8.40	1.60	>0.05
	Spain	8.96	1.60	
Correctly informed	Italy	7.81	1.73	>0.05
	Spain	7.32	1.97	
Self-quarantine (others)	Italy	8.52	2.07	>0.05
	Spain	8.85	1.74	
Self-quarantine (myself)	Italy	8.21	2.09	>0.05
	Spain	8.44	1.99	
Openness	Italy	7.62	1.99	>0.05
	Spain	7.60	1.82	
Extraversion	Italy	6.81	1.64	>0.05
	Spain	6.63	2.06	
Agreeableness	Italy	6.50	1.80	>0.05
	Spain	6.45	1.67	
Conscientiousness	Italy	6.17	1.83	<0.001^a^
	Spain	7.40	1.75	
Neuroticism	Italy	6.40	1.98	>0.05
	Spain	6.08	2.23	

a *Levene's test was statistically significant (p < 0.05), suggesting a violation of the equal variances assumption*.

Secondly, a correlation analysis was carried out under Pearson coefficient employing the whole data (Italy and Spanish samples, Table 2). This procedure was carried out this way, as no differences were found in the previous analysis, except for the Conscientiousness trait of personality.

**Table 2:** Paired Pearson correlations across the variables of interest (whole data).

	**Pearson's** ***r***		**Lower 95% CI**	**Upper 95% CI**
Fear contagious diseases-Sense of belonging	0.46	^***^	0.306	0.594
Fear contagious diseases-Economic impact	0.24	^**^	0.059	0.402
Fear contagious diseases-Correctly informed	0.03		−0.153	0.210
Fear contagious diseases-Self-quarantine (others)	0.21	^*^	0.030	0.378
Fear contagious diseases-Self-quarantine (myself)	0.28	^**^	0.107	0.442
Fear contagious diseases-Openness	−0.18		−0.348	0.004
Fear contagious diseases-Extraversion	−0.15		−0.320	0.036
Fear contagious diseases-Agreeableness	0.07		−0.110	0.251
Fear contagious diseases-Conscientiousness	−0.03		−0.208	0.154
Fear contagious diseases-Neuroticism	0.25	^**^	0.073	0.414
Sense of belonging-Economic impact	0.20	^*^	0.024	0.372
Sense of belonging-correctly informed	0.00		−0.177	0.186
Sense of belonging-Self-quarantine (others)	0.13		−0.057	0.301
Sense of belonging-Self-quarantine (myself)	0.16		−0.019	0.335
Sense of belonging-Openness	−0.18	^*^	−0.354	−0.002
Sense of belonging-Extraversion	−0.16		−0.331	0.023
Sense of belonging-Agreeableness	0.15		−0.031	0.324
Sense of belonging-Conscientiousness	−0.05		−0.234	0.128
Sense of belonging-Neuroticism	0.03		−0.147	0.216
Economic impact-correctly informed	0.21	^*^	0.031	0.379
Economic impact-Self-quarantine (others)	0.31	^***^	0.134	0.464
Economic impact-Self-quarantine (myself)	0.21	^*^	0.029	0.376
Economic impact-Openness	−0.03		−0.206	0.157
Economic impact-Extraversion	−0.08		−0.263	0.098
Economic impact-Agreeableness	0.07		−0.114	0.248
Economic impact-Conscientiousness	0.03		−0.152	0.211
Economic impact-Neuroticism	0.03		−0.151	0.212
Correctly informed-Self-quarantine (others)	0.19	^*^	0.005	0.356
Correctly informed-Self-quarantine (myself)	0.01		−0.168	0.195
Correctly informed-Openness	0.09		−0.096	0.264
Correctly informed-Extraversion	0.09		−0.088	0.272
Correctly informed-Agreeableness	0.11		−0.069	0.289
Correctly informed-Conscientiousness	−0.01		−0.187	0.176
Correctly informed-Neuroticism	−0.13		−0.309	0.048
Self-quarantine (others)-Self-quarantine (myself)	0.73	^***^	0.638	0.808
Self-quarantine (others)-Openness	0.05		−0.132	0.231
Self-quarantine (others)-Extraversion	−0.02		−0.197	0.166
Self-quarantine (others)-Agreeableness	0.01		−0.180	0.183
Self-quarantine (others)-Conscientiousness	0.13		−0.055	0.302
Self-quarantine (others)-Neuroticism	0.03		−0.154	0.209
Self-quarantine (myself)-Openness	−0.15		−0.324	0.031
Self-quarantine (myself)-Extraversion	−0.06		−0.235	0.127
Self-quarantine (myself)-Agreeableness	0.05		−0.135	0.228
Self-quarantine (myself)-Conscientiousness	0.14		−0.037	0.318
Self-quarantine (myself)-Neuroticism	0.07		−0.114	0.248
Openness-Extraversion	0.67	^***^	0.551	0.756
Openness-Agreeableness	0.10		−0.087	0.273
Openness-Conscientiousness	0.19	^*^	0.013	0.363
Openness-Neuroticism	−0.02		−0.198	0.165
Extraversion-Agreeableness	0.09		−0.089	0.271
Extraversion-Conscientiousness	0.19	^*^	0.014	0.364
Extraversion-Neuroticism	−0.01		−0.196	0.167
Agreeableness-Conscientiousness	0.13		−0.055	0.302
Agreeableness-Neuroticism	−0.21	^*^	−0.379	−0.031
Conscientiousness-Neuroticism	−0.05		−0.231	0.131

* *p < 0.05*,

** *p < 0.01*,

*** *p < 0.001*.

**Figure 1:**
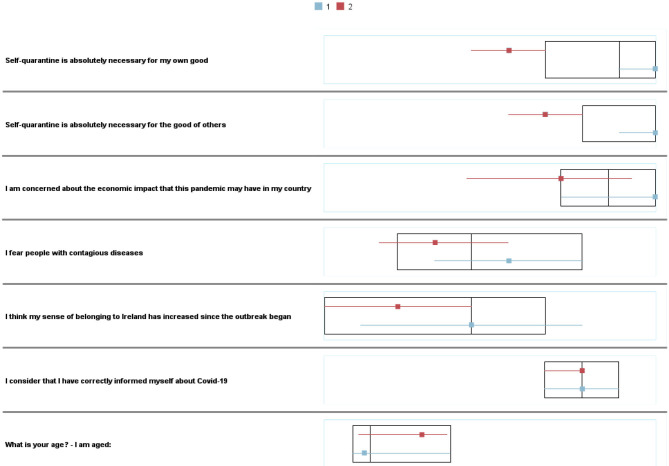
Boxplots on attitudes.

**Figure 2:**
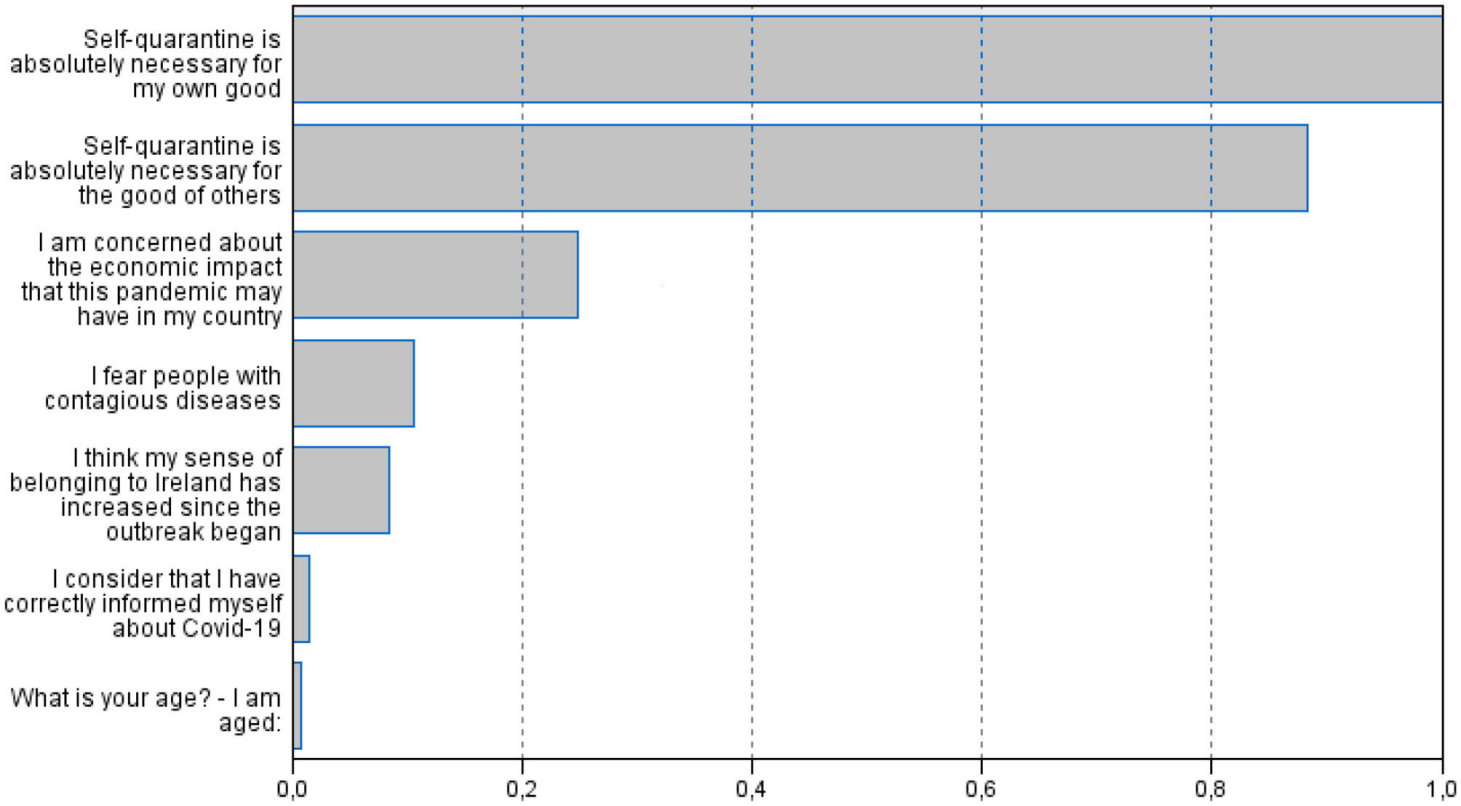
Importance of predictors for the cluster analysis.

A two-step cluster analysis was carried out. Variables of interest, except country and Personality were included in the analysis, and were reanalyzed according to the clusters obtained. Likewise, the Schwarz–Bayesian Inference Criterion (BIC) was employed by using it to select the lowest BIC value in the different estimated models, in this case for two clusters: G_1_ = 81 (72.3%) participants and G_2_= 31 (27.7%) with a ratio of sizes equal to 2.61. Boxplots in Figure 1 depict de differences between the variables of interest to develop the clusters. Figure 2 remarks the relevance of country measures, in this case self-quarantine, as main predictors. As can be seen in Table 3, the clusters differed on only the Neuroticism dimension of personality.

**Table 3:** Independent samples *t*-test after cluster analysis on personality.

	**G_**1**_**	**G_**2**_**	***t***	***p***
Extraversion	6.58 (SD = 1.97)	7.06 (SD = 1.63)	−1.21	0.23
Agreeableness	6.46 (SD = 1.83)	6.41 (SD = 1.47)	0.13	0.89
Conscientiousness	7.22 (SD = 1.89)	6.35 (SD = 1.72)	2.22	0.02
Neuroticism	6.23 (SD = 2.11)	6.09 (SD = 2.27)	0.30	0.76
Openness	7.49 (SD = 1.85)	8.03 (SD = 1.79)	−1.38	0.17

Even if restriction measures were similar among countries, a χ^2^ test was carried out to test the independence between clusters and Countries (*p* = 0.39), and they were classified as follows: 27 Italians plus 54 Spanish participants for G_1_ and 13 Italians plus 18 Spanish participants for G_2_. A *t*-test was employed to examine differences between Clusters in Personality, as depicted in Table 4. Nevertheless, country and clusters reconsidered for a linear regression analysis (Table 4). Thus, a regression linear regression analysis was carried out on the prediction of *Fear Contagious diseases*, which was statistical significant: *F*_(14, 111)_ = 3.83; MSE = 22.30; *p* < 0.001; *R*^2^ = 0.356.

**Table 4:** Coefficients of the predictive model for Fear Contagious diseases after cluster analysis.

**Model**		**B**	**Standard Error**	**β**	***p***
1	(Intercept)	−1.42	3.88		0.71
	People I live with	−0.08	0.09	−0.08	0.33
	Sense of belonging	0.37	0.08	0.39	**<0.0005**
	Economic impact	0.13	0.13	0.08	0.40
	Correctly informed	0.12	0.13	0.08	0.37
	Self-quarantine (others)	−0.04	0.22	−0.03	0.85
	Self-quarantine (myself)	0.18	0.20	0.13	0.36
	Openness	−0.05	0.18	−0.03	0.77
	Extraversion	−0.11	0.16	−0.71	0.48
	Agreeableness	0.10	0.14	0.06	0.49
	Conscientiousness	−0.06	0.15	−0.04	0.77
	Neuroticism	0.29	0.11	0.22	**0.01**
	Age	0.03	0.02	0.18	0.17
	Cluster	−0.27	0.91	−0.30	0.76
	Country	0.246	0.495	0.042	0.61

## Conclusions and Discussion

The aim of this study was to examine the role of personality, country of origin and residence, as well as attitudes confronting death awareness in the Covid-19. For this reason, two samples, from Italy and Spain from participants who have suffer the loss of someone close, were selected. The main results can be described as follows: (i) Personality traits were stable across countries, (ii) A relationship was found between Fear to contagious diseases and Neuroticism and other attitudes during the Covid-19 outbreak, (iii) Two different clusters were identified with regards to attitudes, however these did not differ on Personality, (iv) Cluster group, Neuroticism, Age and Sense of belonging to the Country did predict Fear to contagious diseases.

As expected from previous literature (20), Neuroticism predict Fear in the current samples. This is a robust effect which was also replicated for the current research. Moreover, results seems to support that personality is stable across cluster groups, supporting the idea that invariance across groups occurs, and even in different cultures (25). Nor differences were found for other traits of personality. For this reason, these results also highlight the special psychological attention that people with higher scores in Neuroticism might need during grief.

Of note, no differences were found across countries. One should bear in mind that the type of country restrictions could be comparative in Italy and Spain between March and April, when the study took place. However, it was possible to identify two different profiles across participants, in a homogeneous way across countries. This is an alternative and analytical strategy to traditional analysis (26), that might shed light on common processes across country measures.

On the other hand, attitudes are of interest to understand the effects of Covid-19 outbreak. First, sense of belonging to the country of reference seems to be related and directly predict Fear. This result might support the terror management theory (TMT). In other words, this theory tries to explain how, in times of were death awareness emerge, individuals tend to strengthen their self-esteem to reduce anxiety against death (27). This can also lead to a seek for cohesion between people who share similar world views and hostility toward those with alternative world views (28). Thus, even during process of grief, participants would be susceptible to this effect.

Another variable of interest was to consider yourself well-informed about the Covid-19 virus. Even if the spread of misinformation has been one of the main challenges of the current Covid-19 era (29), no effect were found over this variable. As described in the literature, ambiguous information is related to fear (30). In this population, higher scores were found over their perception, which was correlated with self-quarantine for the others good. This might reflect a special sensibility for the others health. One main limitation over this attitude is related to a self-perception, as the real exposure to media coverage of the Covid-19 crisis was not measured. Future lines of research should examine this perception across real habits and exposures. Nevertheless, we considered it is important to avoid sensational media, which may enhance negative affect, particularly for the grief processes.

Lastly, one might expect this is also related to future situations, such as the Economic impact. However, this variable seems to be related to how well-informed participants perceived themselves, as well as self-quarantine measures. Although quarantine is intended to protect people's health against infectious diseases, restriction of movement can be associated with a variety of psychological problems such as depression, anxiety, fear, loneliness, resentment and confusion (31–33). However, in a population that has suffered a loss of a loved one or close friend, it may also reflect fear of illness, or even have psychological effects beneficial to self-care. In the latter case, remember that it could effectively reduce a person's risk of infection, thus relieving the infectious pressure on the person (34).

We would like to remark some limitations of the current research. First, the sample was selected through a convenience sampling, which can introduce distortions in data though a high component of self (as how well-participants considered they are informed). Secondly, there is a significantly higher number of women than men, which means the results may vary in populations with a greater parity sample, especially considering that this phase of retirement is experienced differently according to gender variable.

## Data Availability Statement

The raw data supporting the conclusions of this article will be made available by the authors, without undue reservation.

## Ethics Statement

The studies involving human participants were reviewed and approved by UCC School of Applied Psychology Ethics Committee on April 6, 2020. The patients/participants provided their written informed consent to participate in this study.

## Author Contributions

MM and CM-T equally contributed to the design and implementation of the research, to the analysis of the results and to the writing of the manuscript.

## Conflict of Interest

The authors declare that the research was conducted in the absence of any commercial or financial relationships that could be construed as a potential conflict of interest.
